# Human Metapneumovirus Infections in Children

**DOI:** 10.3201/eid1401.070251

**Published:** 2008-01

**Authors:** Terho Heikkinen, Riikka Österback, Ville Peltola, Tuomas Jartti, Raija Vainionpää

**Affiliations:** * Turku University Hospital, Turku, Finland; † University of Turku, Turku, Finland

**Keywords:** Human metapneumovirus, children, respiratory tract, otitis media, research

## Abstract

Human metapneumovirus (hMPV) is an important cause of lower respiratory tract infections in hospitalized children, but the age-related incidence and effect of hMPV in unselected children in the community have not been evaluated. We studied a cohort of 1,338 children <13 years of age throughout 1 respiratory season in Finland during 2000–2001. We examined children and obtained a nasal swab for viral detection at any sign of respiratory infection. hMPV was detected in 47 (3.5%) of the 1,338 children. The age-related incidence of hMPV infection was highest (7.6%) in children <2 years of age, in whom hMPV accounted for 1.7% of all infections during the season. During the epidemic peak, hMPV caused 7.1% of all respiratory infections in the cohort. Acute otitis media developed in 61% of hMPV-infected children <3 years of age. Our findings demonstrate that the effect of hMPV in the community is greatest in children <2 years of age.

## Abstract 2

Age-related incidence and effects of these infections are highest among children <2 years of age.

Human metapneumovirus (hMPV) was isolated in 2001 by van den Hoogen et al. in previously virus-negative nasopharyngeal aspirates from children with respiratory tract infections (*1*). Since then, hMPV has been identified wordwide (*2*–*9*). In temperate regions, hMPV circulates mainly during the winter (*6*,*7*,*10*–*12*). Clinical symptoms of hMPV infection resemble those caused by respiratory syncytial virus and range from mild upper respiratory tract infections to wheezing and severe lower respiratory tract illnesses that require hospitalization (*4*,*5*,*10*–*14*). Although hMPV infections have been diagnosed in all age groups, the virus likely has its greatest effect in children (*13*,*14*).

Several studies have demonstrated that hMPV accounts for a major proportion of hospitalizations for lower respiratory tract infections in infants and young children (*10*,*13*,*15*,*16*). The most frequent diagnoses in hospitalized children are bronchiolitis and pneumonia, but occasionally hMPV may also cause severe illnesses that require treatment at intensive care units (*17*,*18*).

Clinical features of hMPV infection in hospitalized children and the role of hMPV as a cause of hospitalization have been well described. However, most children infected with hMPV are treated as outpatients. Although hMPV has been found in substantial numbers of selected outpatient children (*12*,*19*–*21*), to our knowledge, no population-based studies of the incidence and clinical effect of hMPV on unselected children of different ages have been conducted. We determined the incidence, clinical features, and total effect of hMPV infection in a large, prospective, cohort study of respiratory infections in children in Finland.

## Methods

### Study Participants and Study Protocol

This prospective study was conducted in Turku, Finland, from October 9, 2000, through May 20, 2001. The participating children were recruited before the start of the respiratory season in daycare centers, family daycare, and schools in our area (*22*). All children <13 years of age were eligible for participation; no exclusion criteria were used. Of 1,458 children initially enrolled, 1,338 were closely monitored throughout the entire follow-up period. The baseline characteristics of participating children are shown in Table 1.

**Table 1:** Baseline characteristics of 1,338 children at beginning of follow-up, Finland, 2000–2001

Variable	No. children (%)
Age, y	
<1	30 (2.2)
1–<2	219 (16.4)
2–<4	362 (27.1)
4–<6	247 (18.5)
6–<9	232 (17.3)
9–12	248 (18.5)
Sex	
Male	692 (51.7)
Female	646 (48.3)
Child care	
Home or family daycare	261 (19.5)
Daycare center	658 (49.2)
School	419 (31.3)
Diagnosis of asthma	72 (5.4)
Previous wheezing	253 (18.9)
Exposure to tobacco smoke	512 (39.1)*

*Information available for 1,310 children.

Parents were asked to bring their children to the study clinic for examination by a study physician whenever fever or signs of respiratory tract infection appeared. The study clinic was open every day, and all visits were free. In addition to full clinical examination, chest or sinus radiographs were obtained if pneumonia or sinusitis was suspected on the basis of clinical symptoms. Acute otitis media (AOM) was diagnosed by pneumatic otoscopy, aided by routine use of tympanometry and spectral-gradient acoustic reflectometry. Children without any complications at the first visit were reexamined after 5–7 days, or whenever the parents deemed it necessary.

The parents were provided with a symptom diary in which they recorded daily the child’s symptoms and absences from daycare or school and the parents’ absences from work because of the child’s illness. Only actual days lost were recorded. Thus, days of illness occurring on free weekends or other days off were not recorded as causing absenteeism.

### Viral Sampling

At each visit for a new respiratory tract infection, a nasal swab was obtained from a depth of 2–3 cm in the nostril by using a sterile cotton swab that was then inserted into a vial containing viral transport medium (*23*). The specimens were kept in a refrigerator and transported daily to the laboratory at the Department of Virology, University of Turku, where they were subjected to viral culture for influenza viruses, parainfluenza viruses, respiratory syncytial virus, and adenovirus, and PCR assays for rhinoviruses and enteroviruses. The specimens were then frozen at –70°C until thawed for the purposes of this study.

### Detection of hMPV

RNA was extracted from nasal swab specimens by using a High Pure Viral Nucleic Acid Kit (Roche, Basel, Switzerland) according to the manufacturer’s protocol. hMPV was identified in specimens by using reverse transcription–PCR (RT-PCR). Briefly, hMPV was amplified in a 1-step RT-PCR. The RT-PCR mixture contained RT-PCR buffer (50 mmol/L Tris-HCl, pH 8.4, 50 mmol/L NaCl, 4 mmol/L MgCl_2_), 0.5 mmol/L MgCl_2_, 2 mmol/L dithiothreitol, 0.6 mmol/L deoxynucleoside triphosphates, 1 μmol/L of each primer, 20 U Maloney murine leukemia virus reverse transcriptase (Promega, Madison, WI, USA), 4 U Recombinant RNAsin ribonuclease inhibitor (Promega), and 1 U DyNAzyme (Finnzymes, Espoo, Finland). The total volume of the reaction mixture was 50 μL and contained 5 μL of extracted RNA. The RT-PCR for hMPV RNA was conducted at 42°C for 45 min, then at 95°C for 7 min. cDNA amplification consisted of 40 cycles (denaturation at 95°C for 1 min, annealing at 58°C for 1 min, and extension at 72°C for 1 min) and final extension at 72°C for 10 min. Primers for hMPV were from the L gene (*10*), and the forward primer was biotinylated.

Biotinylated hMPV RT-PCR products were detected by using a liquid hybridization assay. In this assay, 10 μL of each RT-PCR product was mixed with 50 μL of DELFIA assay buffer (PerkinElmer Finland Oy, Turku, Finland), added to 3 parallel microtitration wells coated with streptavidin (PerkinElmer Finland Oy), and incubated at room temperature for 30 min. Wells were washed at room temperature with Tris-NaCl buffer containing 0.5% Tween 20, denatured for 5 min with 150 μL denaturation buffer (25 mmol/L NaOH and 5 mmol/L EDTA), hybridized with Eu-labeled hMPV-probe (5′-CTG TTA ATA TCC CAC ACC AG-3′) at 40°C for 2 hours. The hybridization was performed with 100 μL of hybridization solution, in which 2 ng Eu-labeled hMPV probe per well was diluted with DELFIA assay buffer that included 0.85 mmol/L NaCl and 0.1% Tween 20. Unspecific hybridizations were removed with hot (50°C) Tris-NaCl buffer containing 0.5% Tween 20. To enhance fluorescence, 200 μL DELFIA Enhancement solution (PerkinElmer Finland Oy) was added per well and incubated for 10 min with shaking. Fluorescence was measured by using a Victor 1420 Multilabel Counter (PerkinElmer Finland Oy).

### Definitions

AOM was diagnosed by signs of inflammation of the tympanic membrane, the presence of middle ear effusion, and >1 signs of acute infection. The diagnosis of pneumonia was based on radiologic confirmation of the condition. Both complications were associated with hMPV infection if they were diagnosed <14 days after the clinical visit at which the hMPV-positive specimen was obtained.

### Ethics

The study protocol was reviewed and approved by the Ethics Committee of Turku University Hospital. Written informed consent was obtained from the parents of all participating children.

## Results

### hMPV Outbreak

**Figure 1:**
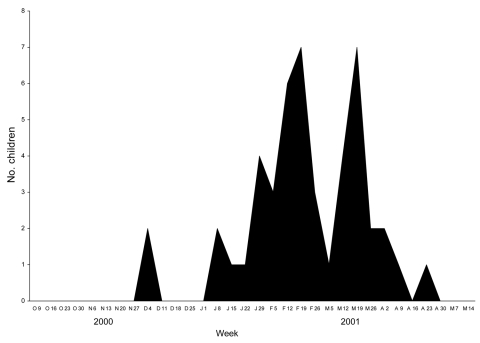
Number of children with human metapneumovirus infections during each week of the study period, Finland, 2000–2001.

The first case of hMPV infection in the study cohort was identified during the week of December 4, 2000, and the last case was identified during the week of April 23, 2001 (Figure 1). hMPV infections were diagnosed weekly over 14 consecutive weeks from January 8, 2001, through April 15, 2001. Overall, hMPV infection was diagnosed in 47 children (26 boys and 21 girls). The median age of the children was 3.0 years; 81% were <5 years of age.

### Incidence Rates of hMPV Infection in Different Age Groups

The incidence of hMPV infection was highest (7.6%) in children <2 years of age at the start of the respiratory season (Table 2). Of a subset of 30 children <1 year of age, 3 (10.0%) acquired hMPV infection during follow-up. The incidence rates of hMPV decreased gradually with age. The overall incidence of hMPV infection in the study cohort of 1,338 children was 3.5% (95% confidence interval [CI] 2.5%–4.5%).

**Table 2:** Incidence of human metapneumovirus (hMPV) infections in children during the respiratory season, Finland, 2000–2001*

Age, y†	No. children	No. children with hMPV	Rate of hMPV/1,000 children (95% CI)
<2	249	19	76 (43–109)
2–<4	362	14	39 (19–59)
4–<6	247	9	36 (13–60)
6–<9	232	3	13 (0–27)
9–12	248	2	8 (0–19)
Total	1,338	47	35 (25–45)

*CI, confidence interval. †Age at beginning of follow-up.

### Relative Effect of hMPV among All Respiratory Infections

The relative proportion of hMPV infections among all respiratory infections was greatest in children <2 years of age, in whom hMPV accounted for 1.7% of all respiratory infections during the winter season (Table 3). During the 14-week period of major hMPV circulation, hMPV accounted for 4.2% of all infections in children <2 years of age. The relative effect of hMPV decreased with age. In the entire cohort of 1,338 children, hMPV accounted for 1.3% (95% CI 0.9%–1.7%) of all respiratory infections during the whole respiratory season and 2.7% (95% CI 1.9%–3.5%) of all infections during the 14-week period of continuous hMPV circulation.

**Table 3:** Proportions of human metapneumovirus (hMPV) infections in children, Finland, 2000–2001

Age, y*	Whole respiratory season			Major hMPV outbreak†	
	Total no. specimens	No. hMPV-positive specimens (%)		Total no. specimens	No. hMPV-positive specimens (%)
<2	1,107	19 (1.7)		430	18 (4.2)
2–<4	1,156	14 (1.2)		518	12 (2.3)
4–<6	631	9 (1.4)		309	9 (2.9)
6–<9	420	3 (0.7)		194	3 (1.5)
9 –12	307	2 (0.7)		151	2 (1.3)
All children	3,621	47 (1.3)		1,602	44 (2.7)

*Age at beginning of follow-up. †Period of 14 consecutive weeks during which hMPV infections were detected every week in the children.

**Figure 2:**
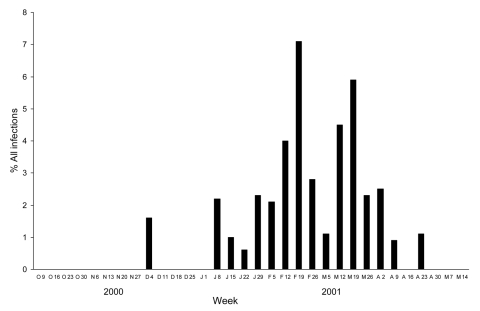
Weekly proportions of human metapneumovirus infections among all respiratory infections in the study children, Finland, 2000–2001.

The proportion of hMPV infections among all respiratory infections during each week of the study is shown in Figure 2. The relative effect of hMPV was highest during the week of February 19, during which the virus accounted for 7.1% of all respiratory infections in the study cohort.

### Co-infections with hMPV

Another virus was detected concomitantly in 8 (17%) of 47 hMPV-infected children. Three children had enterovirus, 2 had rhinovirus, and 1 each had influenza virus, parainfluenza virus, and a nontypeable picornavirus together with hMPV.

### Clinical Characteristics of hMPV Infection

The clinical findings of hMPV infection were analyzed in 39 children in whom hMPV was the only virus detected (Table 4): 97% had a cough, 90% had rhinitis, and 72% had a fever. The median duration of symptomatic illness was 8 days. AOM was the most frequently diagnosed complication; it occurred in 61% of children <3 years of age. Wheezing was observed in 10% of the hMPV-infected children and laryngitis in 8%. A total of 38% of the children were treated with antimicrobial drugs. None of the children was referred to a hospital.

**Table 4:** Clinical characteristics and socioeconomic effects of human metapneumovirus (hMPV) infections in children, Finland, 2000–2001*

Variable	Age group, y†			All children (n = 39)
	1–2 (n = 18)	3–4 (n = 15)	5–9 (n = 6)	
Signs and symptoms				
Cough	18 (100)	15 (100)	5 (83)	38 (97)
Rhinitis	17 (94)	13 (87)	5 (83)	35 (90)
Fever >37.5°C	13 (72)	13 (87)	2 (33)	28 (72)
Vomiting	1 (6)	1 (7)	0	2 (5)
Wheezing	2 (11)	1 (7)	1 (17)	4 (10)
Laryngitis	1 (6)	2 (13)	0	3 (8)
Median duration of illness, d	9.5	8	7	8
Complications				
Acute otitis media	11 (61)	4 (27)	1 (17)	16 (41)
Pneumonia	1 (6)	0	0	1 (3)
Antimicrobial drug treatment	11 (61)	3 (20)	1 (17)	15 (38)
Absenteeism				
Child´s absence from daycare or school for >1 d	10 (56)	8 (53)	3 (50)	21 (54)
Mean duration of child’s absence, d‡	3.7	2.9	3.0	3.3
Parental absence from work for >1 d	9 (50)	4 (27)	2 (33)	15 (38)
Mean duration of parental absence, d‡	2.9	2.5	4.0	2.9

*Values are no. children (%) unless otherwise stated. †Age at diagnosis of hMPV infection. ‡Calculated for children or parents who were absent for at least 1 d.

### Socioeconomic Effect of hMPV

Overall, 54% of hMPV-infected children were absent from daycare or school for >1 day (Table 4). The mean duration of their absence was 3.3 days. In 38% of cases, a parent missed >1 day of work because of the child’s hMPV illness. The average duration of parental work loss was 2.9 days.

### Persistence of hMPV RNA in Nasal Swabs after Acute Illness

In 27 (57%) of the 47 children with hMPV infection, a follow-up nasal swab was obtained during a subsequent respiratory infection. The median interval between the initial hMPV-positive specimen and the subsequent sample was 42 days (range 7–82 days). hMPV could not be detected in any of the subsequent samples after hMPV illness.

## Discussion

This prospective cohort study provides new and detailed information about the effects of hMPV infections in unselected children. Close clinical follow-up of a large cohort of children throughout an entire respiratory season enabled us to determine the incidence and relative importance of hMPV among all respiratory viruses in children in different age groups. A particular strength of our study was that we obtained samples for viral detection during every episode of respiratory illness seen at the study clinic, regardless of the severity of the symptoms or the presence or absence of fever, thereby avoiding any bias caused by sampling only a selected population of children with respiratory infections. Furthermore, symptoms of the children and the socioeconomic effect of their illnesses were recorded daily throughout the study period.

Our findings demonstrate that the effect of hMPV in the community is greatest in the youngest children. The incidence of hMPV infection in children <2 years of age was approximately twice that of children 2–5 years of age and 10× higher than the incidence among children >9 years of age. This finding is consistent with those of serologic studies that demonstrated that most children contract hMPV by 5 years of age (*1*,*24*,*25*). The effect of hMPV on the youngest infants is likely even greater than what was observed in our study. In our cohort, the incidence of hMPV infection was highest in children <1 year of age, but the small number of children in this age group limits our drawing any firm conclusions about this finding.

In addition to the absolute incidence rates of hMPV being highest in children <2 years of age, the relative effect of hMPV among all respiratory viruses was greatest in this age group. During the period of continuous circulation of hMPV, this virus accounted for >4% of all respiratory infections in children <2 years of age. On an annual level, hMPV accounted for 1%–2% of all respiratory infections in our cohort. This estimate agrees with the results of a recent 20-year study in which the prevalence of hMPV ranged from 1% to 5% of all upper respiratory infections in a given year in children <5 years of age (*12*). In our cohort, the overall effect of hMPV was substantially smaller than that of influenza viruses, which accounted for ≈7% of all respiratory infections during the same winter season (*26*). However, the substantial effect of hMPV during local outbreaks is demonstrated by our finding that during the peak of the epidemic, hMPV was responsible for 7% of all respiratory infections in the children even though influenza virus was circulating in the community at the same time (*26*).

Most children with hMPV had cough, rhinitis, and fever. In contrast with previous reports of a high prevalence of wheezing in hospitalized children (*4*,*5*,*7*,*10*,*13*), only 10% of the children seen in primary care had wheezing, and none of these children was referred to a hospital. This is understandable because patients with more severe illnesses usually end up in hospitals, but these results also indicate that most hMPV infections in children are relatively mild and clinically indistinguishable from other viral infections. Of importance, however, is the high rate of AOM as a complication of hMPV infection. Together with similar reports by other investigators (*12*,*13*,*27*), hMPV has a particularly strong ability to predispose a child to AOM. This underscores the clinical similarity between hMPV and respiratory syncytial virus, which is also a major viral cause of AOM in children (*28*–*30*).

In previous studies, hMPV has been infrequently detected in asymptomatic children (*10*,*13*). We could not detect hMPV in any samples obtained during a subsequent respiratory infection after hMPV illness. These findings imply that hMPV in the nasal mucosa is short-lived and corroborate the view that detection of hMPV RNA in respiratory secretions is strongly indicative of a causal role for the virus in the illness.

The variation in prevalence of hMPV from season to season has been demonstrated (*12*,*13*). Therefore, our results obtained during 1 winter season are not directly generalizable to all other winters. However, on the basis of a 2-year hospital-based study in our area, circulation of hMPV was substantially greater during the winter of 2000–2001 than during the following winter (*31*). Thus, our season of follow-up likely did not represent a season with exceptionally low hMPV activity. We obtained nasal swabs instead of nasopharyngeal aspirates for reasons of compliance with repeated sampling. The sensitivity of nasal swabs for detection of various respiratory viruses is ≈90% compared with nasopharyngeal aspirates (*23*). Therefore, some cases of hMPV may not have been diagnosed. Furthermore, because we did not use serologic analysis, we have no data on the incidence of hMPV infections that were either asymptomatic or so mild that the parents did not bring the children to the study clinic. However, the clinical importance of such subclinical infections is negligible.

In conclusion, our prospective population-based study provides conclusive evidence for the effect of hMPV infections in children. The effect of hMPV is greatest in children <2 years of age. hMPV also appears to be one of the major viruses predisposing children to AOM. Although on an annual level hMPV accounts only for a small proportion of all respiratory infections in children, its relative role among all respiratory viruses is substantial during local hMPV outbreaks.
